# Targeting PKGIα Cys^42^ attenuates cardiac dysfunction in heart failure with preserved ejection fraction

**DOI:** 10.1126/sciadv.aec8088

**Published:** 2026-08-19

**Authors:** Jie Su, Yue Zhao, Pierre Coleman, Xiaoping Yang, Mark Holt, Janice Raabe, Friederike Cuello, Ajay Shah, Michael J. Shattock, Min Zhang, Joseph R. Burgoyne

**Affiliations:** ^1^ School of Cardiovascular and Metabolic Medicine & Sciences, King’s College London, The British Heart Foundation Centre of Excellence, The Rayne Institute, St Thomas’ Hospital, London SE1 7EH, UK.; ^2^ King’s College London, Proteomics Facility, Centre of Excellence for Mass Spectrometry, The James Black Centre, 125 Coldharbour Lane, London SE5 9NU, UK.; ^3^ School of Cardiovascular and Metabolic Medicine & Sciences, King’s College London, The British Heart Foundation Centre of Excellence, Randall Centre for Cell and Molecular Biophysics, New Hunt’s House, London SE1 1UL, UK.; ^4^ Institute of Experimental Pharmacology and Toxicology, Cardiovascular Research Center, University Medical Center Hamburg-Eppendorf, Martinistrasse 52, 20246 Hamburg, Germany.; ^5^ School of Cardiovascular and Metabolic Medicine & Sciences, King’s College London, The British Heart Foundation Centre of Excellence, The James Black Centre, 125 Coldharbour Lane, London SE5 9NU, UK.

## Abstract

Heart failure with preserved ejection fraction (HFpEF) is a highly prevalent condition associated with substantial morbidity and mortality, yet effective therapeutic options remain limited. As oxidation of Cys^42^ in cyclic guanosine monophosphate (cGMP)-dependent protein kinase Iα (PKGIα) can enhance vessel and diastolic relaxation, processes impaired in HFpEF, we sought to target this mechanism using natural compounds with predicted thiol reactivity. Among these, urolithin A emerged as a compound with a previously unidentified and counterintuitive mode of action, directly modifying Cys^42^ in PKGIα. In a multihit HFpEF model that closely mimics the human condition, urolithin A improved diastolic function and attenuated cardiac remodeling through cysteine 42–dependent activation of PKGIα. These findings were further validated in human engineered heart tissue, where urolithin A enhanced both relaxation and contraction kinetics. These findings collectively highlight Cys^42^ in PKGIα as a promising therapeutic target for HFpEF and identify urolithin A as a previously unidentified activator of this protective mechanism.

## INTRODUCTION

Heart failure with preserved ejection fraction (HFpEF) accounts for nearly half of all heart failure cases and is associated with substantial morbidity and mortality (*1*). This prevalent condition is challenging to treat due to its complex pathophysiology, which is predominantly characterized by diastolic dysfunction. This dysfunction impairs ventricular relaxation and filling, often exacerbated during physical exertion, highlighting a limited cardiovascular reserve capacity, particularly in older individuals.

The cyclic guanosine monophosphate (cGMP)-dependent protein kinase Iα (PKGIα) plays a critical role in cardiovascular health via its activation by the canonical nitric oxide (NO)/cGMP pathway or through alternative mechanisms involving oxidants or 2′,3′-cyclic GMP-AMP (cGAMP) (*2*, *3*). Oxidation of cysteine 42 (C42) on PKGIα by reactive oxygen species (ROS) or electrophilic compounds promotes disulfide dimerization (*3*, *4*), a process essential for regulating blood pressure (*5*, *6*), and facilitating diastolic relaxation during myocardial stretch by enhancing phospholamban (PLN) phosphorylation (*7*). On the basis of these established functions, we hypothesized that C42 on PKGIα may represent a previously unidentified therapeutic target to limit the progression of HFpEF.

Although natural polyphenols are often regarded as antioxidants due to their ROS-scavenging properties (*8*), this view is oversimplified. Because of their redox cycling properties, polyphenols can paradoxically act as prooxidants by modifying protein cysteine thiols through Michael addition reactions (*9*). This includes the formation of reactive quinones, which directly modify cysteine residues, and the generation of superoxide. Such modifications may underlie some beneficial effects of polyphenols. For instance, our previous research demonstrated that resveratrol mediates C42-dependent activation of PKGIα, which lowers blood pressure in hypertensive mice (*3*).

In this study, we identified several polyphenols, including urolithin A, that have a previously unidentified counterintuitive mechanism of action by potently inducing C42-dependent activation of PKGIα. To evaluate the therapeutic potential of this mechanism, we used a multihit HFpEF model that closely mimics the human condition (*10*). In this model, treatment with urolithin A substantially improved diastolic function and attenuated cardiac remodeling, with both effects shown to be dependent on C42-mediated activation of PKGIα. In addition, urolithin A enhanced relaxation and contraction kinetics in human engineered heart tissue (EHT), highlighting its translational potential.

## RESULTS

### Quercetin and fisetin mediate PKGIα C42 dimerization and enhance substrate phosphorylation

**Fig. 1.**
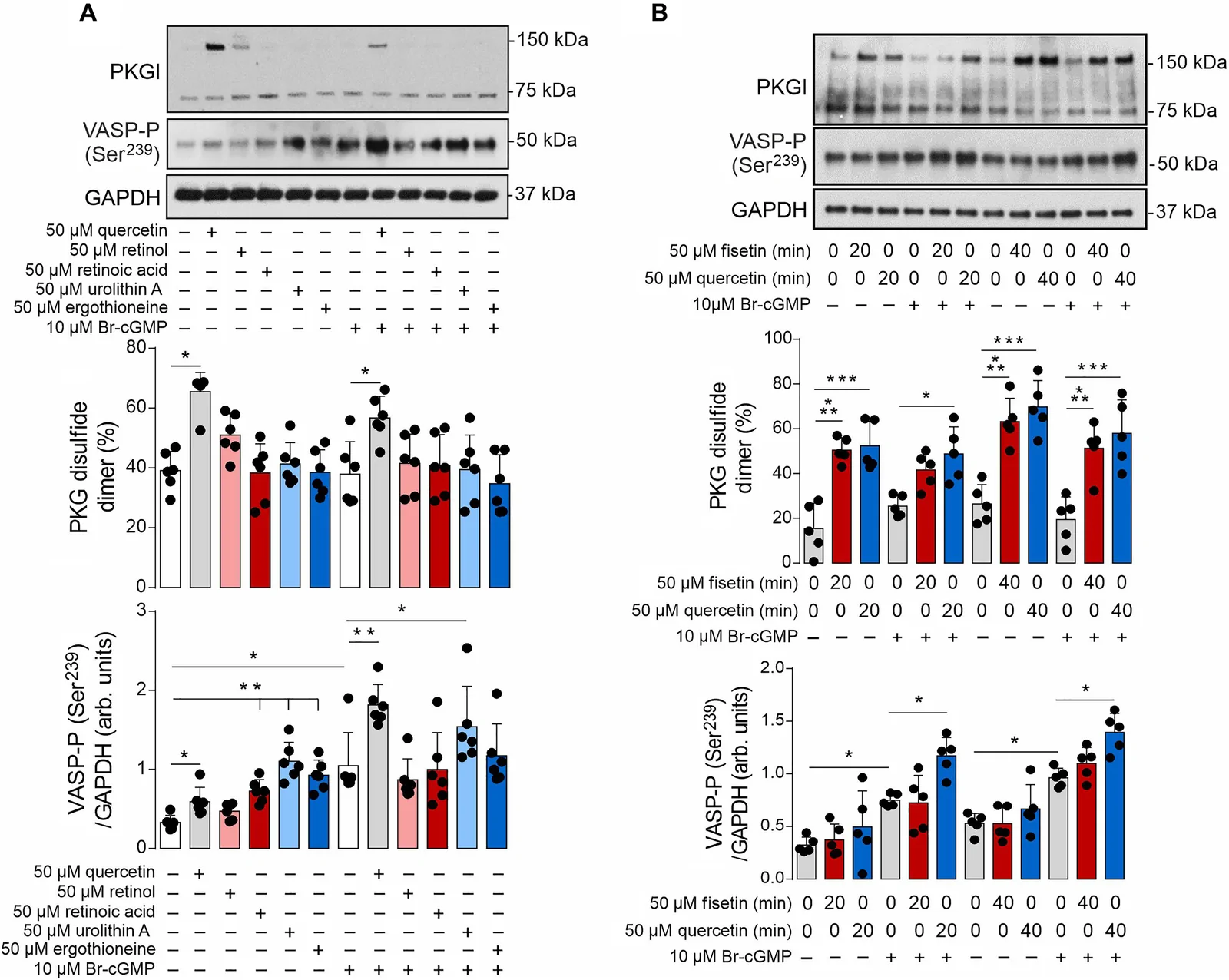
Fisetin and quercetin mediate PKGIα disulfide dimerization and increase substrate phosphorylation. (**A**) PKGIα disulfide dimerization and VASP Ser^239^ phosphorylation in vascular smooth muscle cells treated with quercetin, retinol, retinoic acid, urolithin A, or ergothioneine in the presence or absence of Br-cGMP (*n* = 6). (**B**) PKGIα disulfide dimerization and VASP Ser^239^ phosphorylation in vascular smooth muscle cells treated with fisetin or quercetin in the presence or absence of Br-cGMP (*n* = 5). *P < 0.05; **P < 0.01; ***P < 0.005. Comparisons were made using one-way ANOVA followed by the Tukey post hoc test.

Natural compounds with potential thiol reactivity were evaluated for their ability to induce PKGIα disulfide dimerization and enhance kinase activity (Fig. 1A). Among the compounds tested, only quercetin effectively induced PKGIα disulfide dimerization. Consistent with this, quercetin also promoted substrate phosphorylation and synergized with cGMP to enhance kinase activation. Although retinoic acid, urolithin A, and ergothioneine did not induce PKGIα disulfide dimerization, they still enhanced substrate phosphorylation. Of these, only urolithin A synergized with cGMP, suggesting that it may activate PKGIα through a noncanonical mechanism.

Given the effectiveness of quercetin in inducing PKGIα disulfide dimerization, its activity was compared with the structurally similar flavonoid fisetin. Both compounds induced comparable PKGIα disulfide dimerization (Fig. 1B) and synergized with cGMP to enhance vasodilator-stimulated phosphoprotein (VASP) phosphorylation, consistent with C42-dependent kinase activation. In line with its thiol reactivity, quercetin also induced direct cysteine oxidation (fig. S1).

### Urolithin A mediates C42-dependent PKGIα activation and vessel dilation

**Fig. 2.**
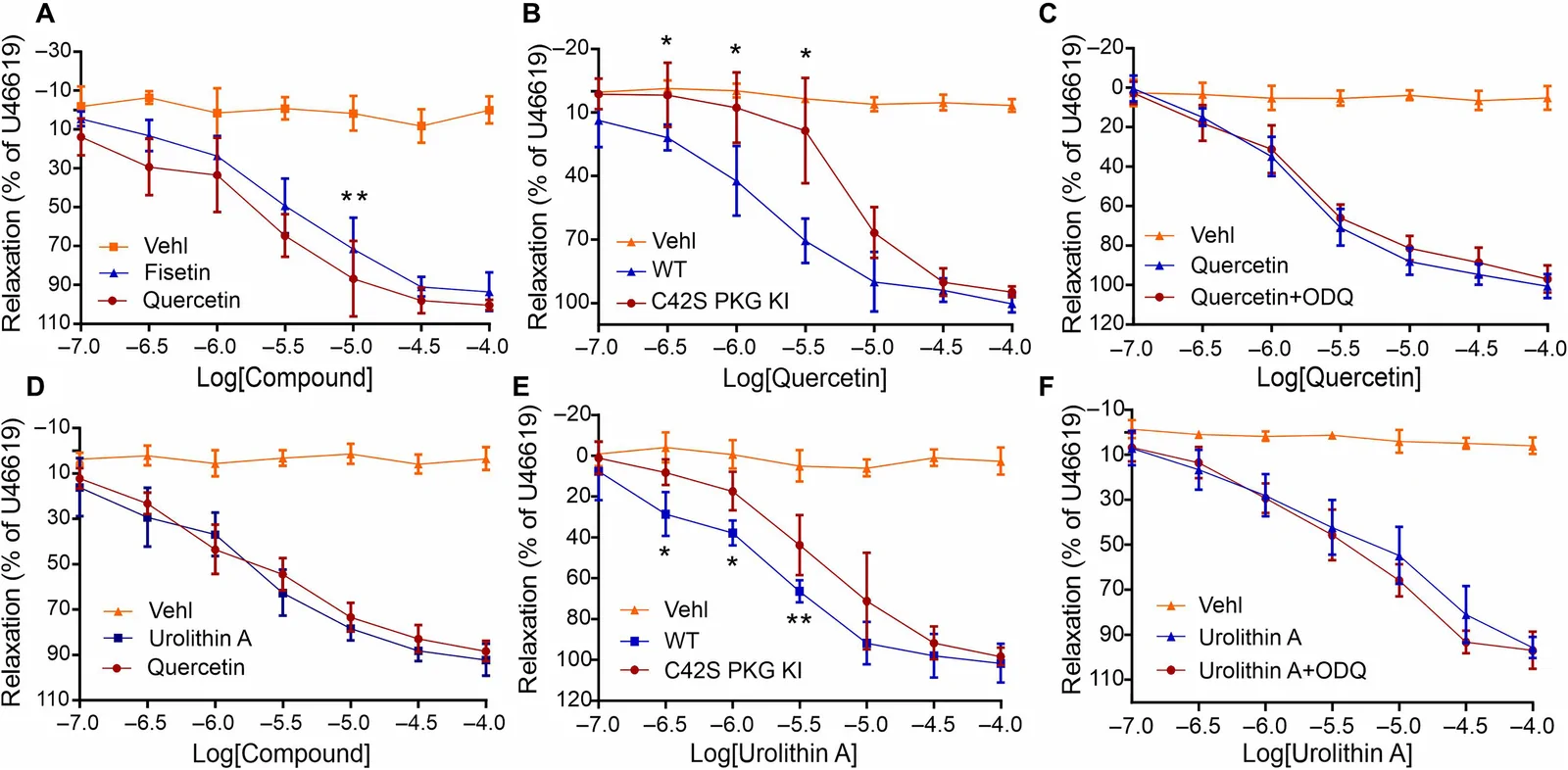
Quercetin and urolithin A induce PKGIα C42-dependent vessel relaxation. (**A**) Comparing mesenteric vessel relaxation to quercetin or fisetin (*n* = 5). (**B**) Assessing relaxation of mesenteric vessels from WT and C42S PKG KI mice to quercetin (*n* = 4). (**C**) Assessing the impact of sGC inhibition on mesenteric vessel relaxation to quercetin (*n* = 5). (**D**) Comparing mesenteric vessel relaxation to quercetin or urolithin A (*n* = 4). (**E**) Comparing relaxation of mesenteric vessels from WT and C42S PKG KI mice to urolithin A (*n* = 6). (**F**) Assessing the impact of sGC inhibition on mesenteric vessel relaxation to urolithin A (*n* = 5). *P < 0.05; **P < 0.01. Comparisons were made using two-way ANOVA followed by the Tukey post hoc test.

We next assessed the ability of quercetin and fisetin to modulate vasotone. Consistent with oxidative PKGIα activation, both compounds induced vasodilation (Fig. 2A). Quercetin was more effective in inducing vasodilation, which was found to be in part dependent on C42 in PKGIα as this was blunted in mesenteric vessels isolated from PKGIα cysteine 42 to serine (C42S) knock-in (KI) mice (Fig. 2B). To further validate the mechanisms involved, soluble guanylate cyclase (sGC) was inhibited using ODQ. Consistent with C42-dependent PKGIα activation, inhibition of sGC did not affect relaxation to quercetin (Fig. 2C).

Although urolithin A did not induce notable PKGIα disulfide dimerization, it still synergized with cGMP-dependent kinase activation, suggesting the potential for C42-dependent kinase activation. Consistent with PKGIα activation, urolithin A mediated mesenteric vessel relaxation that was comparable to quercetin (Fig. 2D). Furthermore, the relaxation mediated by urolithin A was attenuated in vessels from KI mice (Fig. 2E). This suggests that, despite the lack of intermolecular disulfide formation, urolithin A likely activates PKGIα through oxidation of C42. This was further supported by experiments where sGC inhibition did not prevent relaxation by urolithin A (Fig. 2F).

### Urolithin A directly activates PKGIα, which leads to enhanced cardiomyocyte PLN phosphorylation

**Fig. 3.**
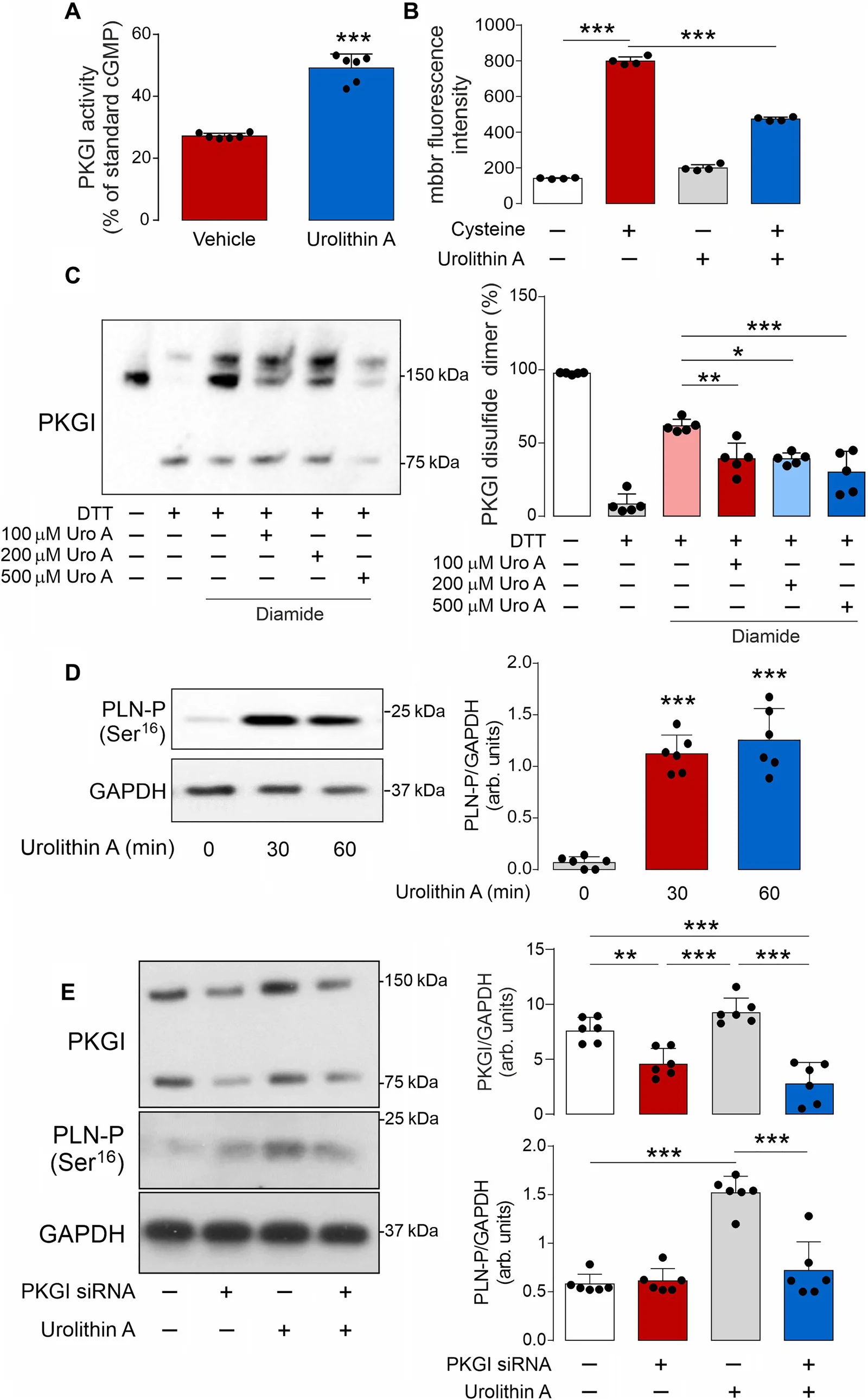
Urolithin A induces direct oxidation of PKGIα C42 to mediate kinase activation and enhance cardiomyocyte phospholamban phosphorylation. (**A**) The activity of recombinant PKGIα in the presence or absence of urolithin A (*n* = 6). (**B**) Monobromobimane (mBBr) assay assessing the modification of cysteine by urolithin A (*n* = 4). (**C**) Treatment of reduced recombinant PKGIα with diamide in the presence or absence of urolithin A (*n* = 5). (**D**) Assessing PLN Ser^16^ phosphorylation in cardiomyocytes treated with or without urolithin A (*n* = 6). (**E**) Assessing the impact of knocking down PKGI on urolithin A–dependent PLN Ser^16^ phosphorylation in cardiomyocytes (*n* = 6). *P < 0.05; **P < 0.01; ***P < 0.005. Comparisons were made using one-way ANOVA [(A), (C), and (D)] or two-way ANOVA [(B) and (E)] followed by the Tukey post hoc test.

Given urolithin A was able to induce vessel relaxation that was dependent on C42 in PKGIα, but independent of intermolecular disulfide formation, we further investigated the mechanism involved. In in vitro kinase assays, urolithin A directly activated PKGIα, consistent with oxidation-dependent kinase stimulation (Fig. 3A). This finding was corroborated by assays measuring the thiol reactivity of urolithin A, which demonstrated its ability to compete with the alkylating agent monobromobimane (mBBr; Fig. 3B). As urolithin A was likely to stimulate PKGIα through oxidation of C42, we hypothesized that this compound was modifying this residue to mimic the action of disulfide formation. This was substantiated in experiments where urolithin A limited the disulfide dimerization of PKGIα to diamide and led to a urolithin A adduct on C42 detected by mass spectrometry (Fig. 3C and fig. S2). Because C42 oxidation on PKGIα is linked to enhanced cardiac myocyte relaxation through PLN phosphorylation (*7*), we next evaluated whether urolithin A could also mediate this process. Here, we found that urolithin A significantly increased cardiomyocyte PLN phosphorylation (Fig. 3D). Consistent with this being dependent on PKGIα activation, knockdown of this kinase attenuated PLN phosphorylation in cardiomyocytes treated with urolithin A (Fig. 3E).

### PKGIα C42 oxidation by urolithin A limits diastolic dysfunction and cardiac remodeling

**Fig. 4.**
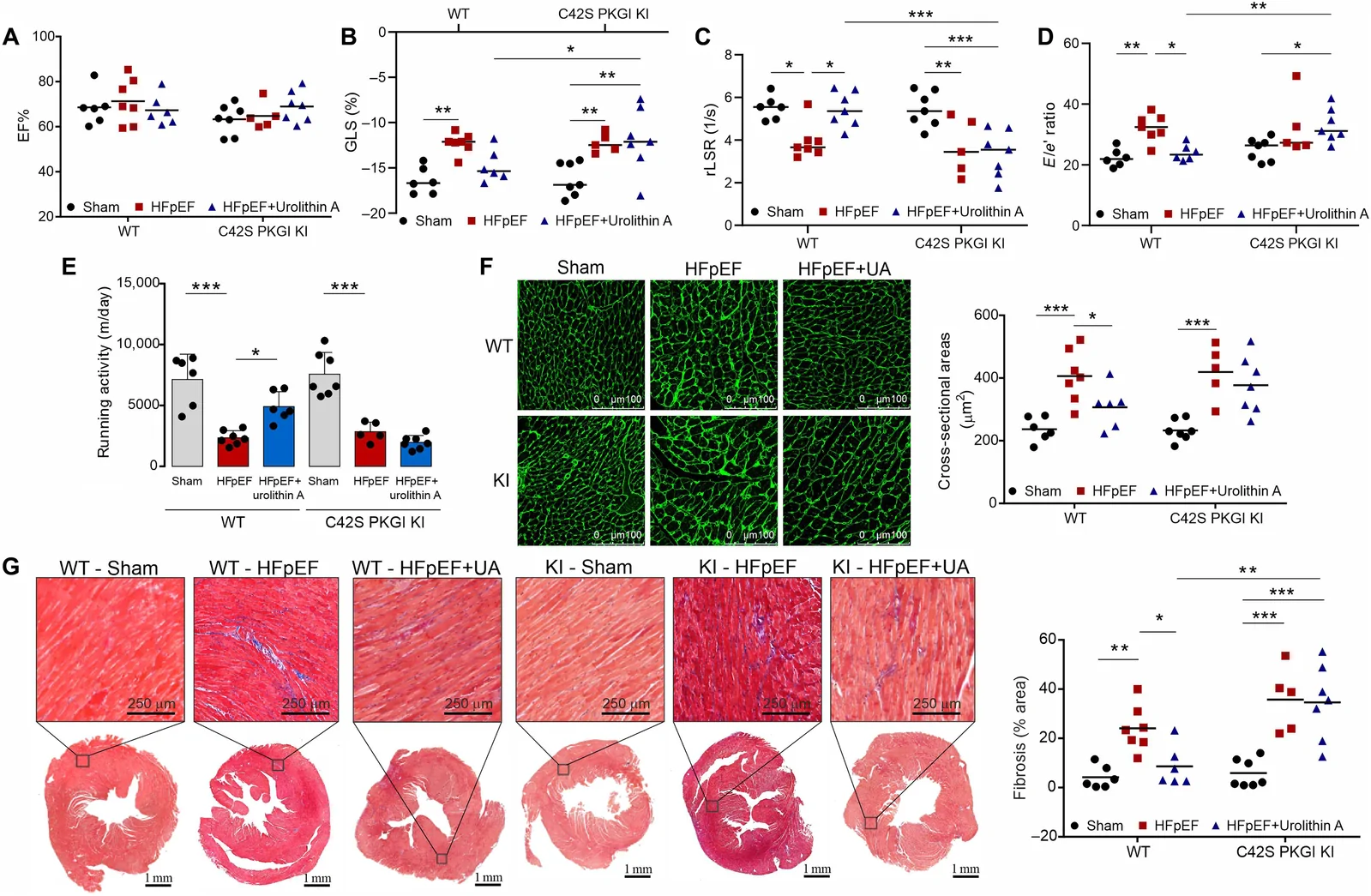
Urolithin A limits HFpEF that is dependent on PKGIα C42. Assessing (**A**) left ventricular ejection fraction (EF), (**B**) global longitudinal strain (GLS), (**C**) reverse longitudinal strain rate (rLSR), (**D**) *E*/*e*′ ratio, and (**E**) running activity in WT and C42S PKGIα KI mice after sham surgery, HFpEF, of HFpEF with 1 week of diet supplementation with urolithin A (*n* = 5 to 7). Assessing (**F**) myocyte cross-sectional area and (**G**) fibrosis in heart tissue excised from WT or C42S PKGIα KI mice after sham surgery, HFpEF, of HFpEF with 1 week of diet supplementation with urolithin A (*n* = 5 to 7). *P < 0.05; **P < 0.01; ***P < 0.005. Comparisons were made using two-way ANOVA followed by the Tukey post hoc test.

Given the therapeutic potential of urolithin A, including bioavailability and safety profile in humans (*11*–*14*), we evaluated the impact of this compound on myocardial dysfunction in HFpEF. Here, we used a multihit preclinical model where mice were subjected to nephrectomy and treated with 11-deoxycorticosterone acetate (DOCA)–salt and a high-fat (HF) diet, as previously described (*10*). This combination of metabolic and hypertensive stress reproduced key cardiovascular features of human HFpEF (Fig. 4, A to G, and fig. S3, A to H). To investigate the role of PKGIα C42, HFpEF was induced in both wild-type (WT) and KI mice, which have been extensively characterized (*3*–*7*). After HFpEF was established, mice were orally administered either vehicle or urolithin A for 7 days. Echocardiography demonstrated that left ventricular ejection fraction (EF; Fig. 4A) and fractional shortening (FS; fig. S3A) were preserved across groups, with no detectable effect of urolithin A on systolic function or heart rate (fig. S3B). In contrast, WT mice treated with urolithin A exhibited significant improvements in global longitudinal strain (GLS; Fig. 4B), average reverse longitudinal strain rate (rLSR; Fig. 4C), and *E*/*e*′ ratio (Fig. 4D), indicating enhanced diastolic performance following treatment. Additional indices, including the *E*/*A* ratio (fig. S3C), were also improved, although heart rate–corrected isovolumetric relaxation time (IVRTc; fig. S3D) remained unchanged. Concordantly, left ventricular mass and heart weight were reduced in urolithin A–treated mice (fig. S3, E to G), further supporting a structural and functional benefit. These improvements in cardiac function and structure were accompanied by an increase in running distance (Fig. 4E and fig. S3H), as well as a reduction in fibrosis, cardiomyocyte cell size, and atrial natriuretic peptide (ANP) expression (Fig. 4, F and G, and fig. S3I). Although PKGIα disulfide dimerization was not altered during HFpEF (fig. S3J), the beneficial effects of urolithin A were dependent on PKGIα C42, consistent with myocardial kinase activation that was absent in KI mice (fig. S3K). Thus, urolithin A limits cardiac dysfunction and remodeling in HFpEF through C42-dependent PKGIα activation.

### Urolithin A improves the kinetics of relaxation and contraction in human EHT

**Fig. 5.**
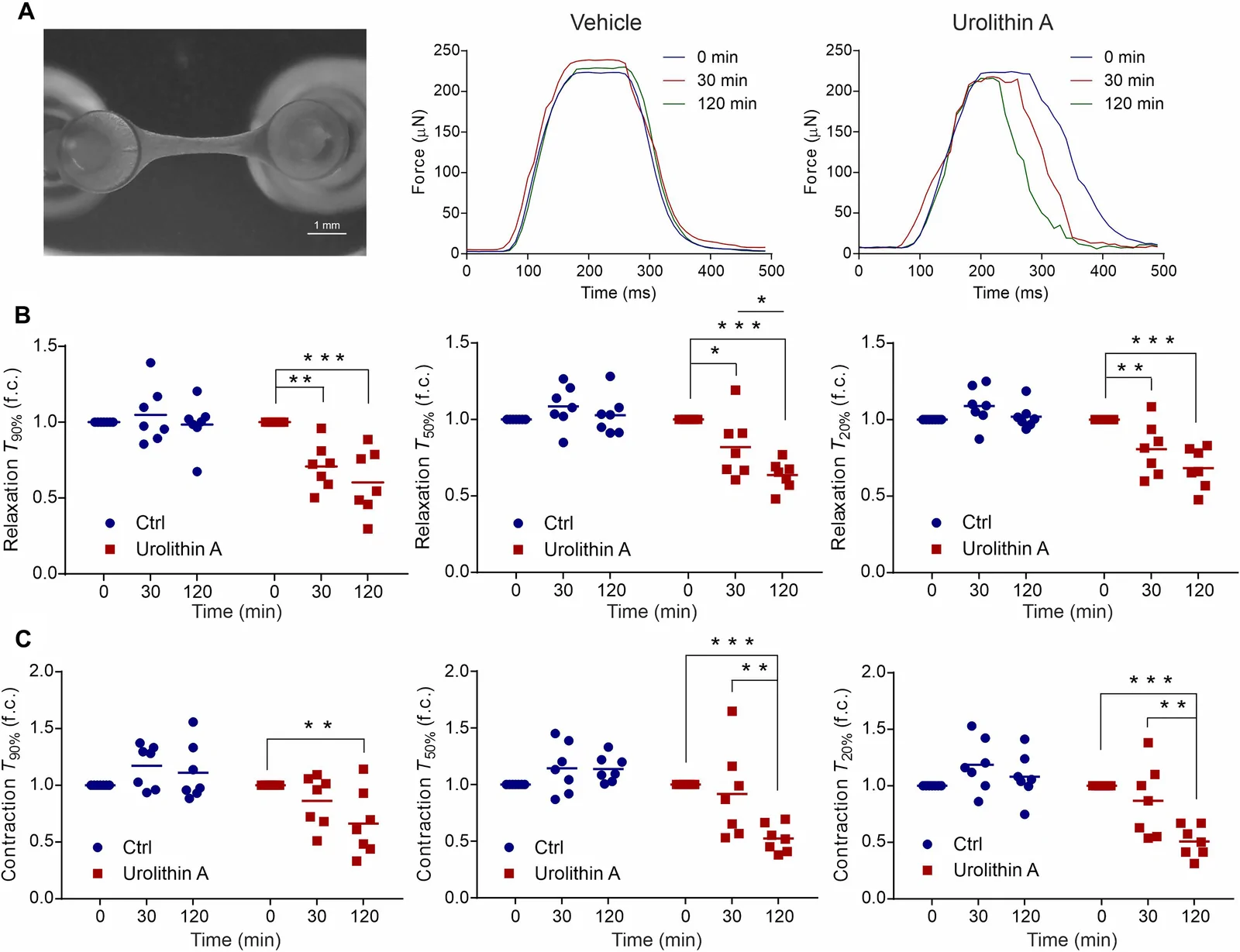
Urolithin A improves relaxation and contraction kinetics in engineered human heart tissue. (**A**) Representative force generation in EHTs treated with vehicle or urolithin A. (**B**) Kinetics of relaxation in EHTs treated with or without urolithin A (*n* = 6). (**C**) Kinetics of contraction in EHTs treated with vehicle or urolithin A (*n* = 6). *P < 0.05; **P < 0.01; ***P < 0.005. Comparisons were made using two-way ANOVA followed by the Tukey post hoc test.

To further evaluate the potential of urolithin A for treating HFpEF, we assessed its effects on contractile function in human EHT derived from cardiomyocytes generated from a single donor human induced pluripotent stem cell line. In tissues treated with urolithin A, there were no changes in maximal force generation or arrhythmias detected (Fig. 5A). However, there was a significant improvement in the contraction and relaxation times. Parameters of relaxation were decreased (with 120 min urolithin A treatment, *T*_20%_ by 32%, *T*_50%_ by 36%, and *T*_90%_ by 40%; Fig. 5B) as were measures of contraction (with 120 min urolithin A treatment, *T*_20%_ by 49%, *T*_50%_ by 48%, and *T*_90%_ by 34%; Fig. 5C). This is consistent with improved Ca^2+^ handling, likely by polyphenol-induced PKGI-dependent phosphorylation of PLN (*7*). Together, these findings suggest that urolithin A can enhance contraction and relaxation parameters in human cardiac tissue.

## DISCUSSION

Impaired NO bioavailability and reduced PKGI activity are associated with the pathology of HFpEF, as supported by clinical evidence (*15*–*17*). On the basis of this understanding, therapeutic strategies targeting this pathway have been explored using the phosphodiesterase type 5 (PDE5) inhibitor sildenafil and soluble guanylyl cyclase (sGC) stimulator vericiguat (*18*, *19*). However, these approaches failed to improve the primary outcomes or secondary endpoints in clinical trials. Several factors may explain these outcomes. Endothelial dysfunction in patients with HFpEF likely results in reduced NO and natriuretic peptide bioavailability (*20*, *21*), diminishing the efficacy of PDE5 inhibition. In addition, the VITALITY trial using vericiguat may have included a less symptomatic HFpEF population, which could have influenced the results. Alternatively, it is possible that NO signaling is not central to the progression of HFpEF, a hypothesis supported by the failure of oral nitrates and inhaled nitrites to improve clinical outcomes in this population (*22*, *23*). Our previous work demonstrated that cGMP mitigates C42-dependent PKGIα activation (*24*), a process known to enhance diastolic relaxation (*7*). This suggests that the limited efficacy of NO-targeted therapies may arise from mechanisms beyond impaired NO signaling. Consequently, we hypothesized that directly targeting C42-dependent activation of PKGIα could alleviate HFpEF progression by mediating vasodilation while directly improving diastolic function. This rationale led to the identification of a previously unidentified therapeutic strategy for HFpEF by targeting C42 in PKGIα using urolithin A.

Several natural compounds were selected for their potential to activate PKGIα via C42 thiol oxidation. These compounds were chosen based on their intrinsic α,β-unsaturated carbonyl groups or their ability to form such modifications through redox cycling. The presence of an α,β-unsaturated carbonyl group provides a capacity to modify cysteine thiols through a Michael addition reaction (*25*). Among the selected compounds, quercetin and its structurally similar flavonol, fisetin, were particularly effective in inducing PKGIα disulfide dimerization and enhancing kinase activation. In addition, urolithin A, which did not promote intermolecular C42 disulfide formation, significantly enhanced PKGIα activation and demonstrated synergy with cGMP. This finding highlighted the potential for urolithin A to also mediate redox-dependent kinase activation. This aligns with our previous findings, where the C42-dependent PKGIα activator G1 also synergized with cGMP (*6*). The likely mechanism for this synergy involves C42 oxidation, which enhances substrate interaction, while cGMP directly activates the kinase.

Consistent with the synergistic activation of PKGIα, both quercetin and urolithin A were able to induce vasodilation that was dependent on C42. As urolithin A did not induce notable detection of the C42 PKGIα intermolecular disulfide, this indicated a different mechanism of kinase activation. It was rationalized that this was likely through formation of a urolithin A adduct on C42. Consistent with the adduction of urolithin A on C42, this compound was found to be thiol reactive and could induce direct PKGIα activation. Furthermore, urolithin A limited the disulfide dimerization of PKGIα mediated by diamide, thus consistent with a urolithin A adduct on C42, which was confirmed by mass spectrometry analysis. It is likely that urolithin A is able to adduct to C42 through redox cycling and formation of a thiol-reactive quinone intermediate.

Given its bioavailability, favorable pharmacokinetic profile, and the health benefits associated with oral consumption of urolithin A in humans, this compound was further explored as a potential therapy for HFpEF (*11*–*13*). In humans, urolithin A has a favorable safety profile, and its bioavailability has been shown to improve mitochondrial and cellular health in elderly individuals (*12*). In this study, urolithin A was found to be bioavailable at all tested doses (250 to 2000 mg) and had a relatively long half-life ranging from 17 to 22 hours. In addition, in humans administered a single dose of pomegranate juice or pomegranate extract, plasma urolithin concentrations exceeded 2.0 μM (470 ng/ml) at 24 hours and remained elevated through 48 hours (*26*). In addition, a study found that urolithin A supplementation was safe and well tolerated and that long-term exposure likely counteracts age-associated muscle decline by improving muscle endurance and plasma biomarkers (*13*). In obese mice, oral administration of urolithin A alleviated metabolic cardiomyopathy by activating mitophagy (*27*). In mice, oral administration of urolithin A has also been shown to limit muscle dysfunction (*28*), Alzheimer’s disease (*29*), multiple sclerosis (*30*), colitis (*31*), and cancer (*32*), consistent with its systemic bioavailability.

In cardiomyocytes, urolithin A induced PKGIα-dependent phosphorylation of PLN. This is consistent with the preferential phosphorylation of PLN by the C42-oxidized form of PKGIα (*7*). Phosphorylation of PLN at Ser^16^ relieves its inhibitory effect on SERCA2a, enhancing the uptake of Ca^2+^ into the sarcoplasmic reticulum (*33*). This mechanism of Ca^2+^ regulation by PKGIα contributes to diastolic relaxation and is involved in myocardial stretch, where it fine-tunes the Frank-Starling response (*7*). Consistent with the ability of urolithin A to modulate PKGIα through C42 oxidation, this compound was found to limit the severity of HFpEF. In addition, using redox-dead C42S KI PKGIα mice, we confirmed that the beneficial effects of urolithin A were primarily mediated through the oxidation of this residue. It is likely C42 thiol-dependent activation of PKGIα provides a dual benefit as it will not only mediate vasodilation but also directly enhance diastolic function. Together, these mechanisms limit cardiac remodeling and dysfunction. This is consistent with the well-established role of PKGIα C42 in mediating blood pressure lowering (*3*, *5*), as well as in regulating SERCA2a activation (*7*). Given the potential for urolithin A to also limit inflammation and enhance mitophagy, these processes may also contribute to the beneficial actions of this polyphenol to limit HFpEF (*34*). Furthermore, the mechanism of PKGIα activation by urolithin A differs from intermolecular disulfide formation, as this polyphenol was found to directly adduct to C42. Direct modification of this residue is likely to perturb the neighboring autoinhibitory region that regulates protein function via a pseudosubstrate sequence (*35*). Accordingly, directly targeting C42 in PKGIα, either with urolithin A or through the development of more potent and selective thiol-reactive compounds, may represent a previously unidentified therapeutic strategy for treating HFpEF.

We established a multihit HFpEF model by integrating mineralocorticoid-driven hypertension (DOCA-salt/uninephrectomy) with metabolic stress induced by HF diet. DOCA-salt promotes sustained blood pressure elevation through aldosterone-mediated mineralocorticoid receptor activation (*36*–*38*), driving sodium retention, vascular dysfunction, oxidative stress, and myocardial remodeling pathways central to HFpEF pathogenesis (*39*) and clinically validated by mineralocorticoid receptor antagonism (*40*). In combination with metabolic challenge, this approach rapidly produced a robust early-stage HFpEF phenotype characterized by preserved EF alongside reduced GLS, impaired rLSR indicative of defective active relaxation, decreased exercise capacity, and increased interstitial fibrosis. The early emergence of deformation abnormalities suggests subendocardial and lusitropic dysfunction preceding extensive extracellular matrix expansion, consistent with the proposed temporal progression of HFpEF remodeling. Reproducibility of this phenotype in an independent facility and a different mouse background (*10*) further supports the robustness and translational relevance of this model for mechanistic interrogation of diastolic dysfunction. Although elevated *E*/*e*′ and reduced *E*/*A* ratios were observed and improved following urolithin A treatment, transmitral Doppler parameters in mice must be interpreted cautiously given the extreme heart rate and compressed diastolic intervals relative to humans (*41*, *42*), making deformation-based indices mechanistically more informative in this setting.

The rapidly reversed key features of HFpEF by urolithin A mirrored findings from studies using the iNOS inhibitor l-NIL, which improved ventricular relaxation and exercise performance after 3 days of treatment in mice subjected to HF diet and l-NAME administration (*43*). The beneficial effects of targeting PKGIα in HFpEF are not observed in HFrEF, where this mechanism has instead been implicated in disease pathogenesis (*44*). This difference likely reflects the distinct underlying processes that govern the development and pathology of HFpEF compared with HFrEF.

In the present study, we focused on male mice to establish a well-controlled HFpEF model. Although human HFpEF disproportionately affects women, highlighting a clear sex bias in prevalence and phenotype, preclinical studies have shown sex-dependent differences, with male and female rodents exhibiting distinct cardiac responses to metabolic and hypertensive stress (*45*–*47*). Consequently, whether the present model accurately recapitulates the HFpEF phenotype in females remains to be addressed in future investigations.

To evaluate the translational potential of urolithin A in humans, we assessed its impact on the functional parameters of engineered human heart tissue. Urolithin A was found to improve the kinetics of both relaxation and contraction. This is consistent with improved Ca^2+^ handling, likely through urolithin A–induced PKGI-dependent phosphorylation of PLN (*7*). Together with these findings, our results identify urolithin A as a previously unidentified thiol-dependent activator of PKGIα, which limits the severity of HFpEF in a preclinical model and is likely to improve cardiac function in patients with this condition.

## MATERIALS AND METHODS

### Isolation and culture of rat aortic smooth muscle cells

The thoracic aortas of rats (male Sprague-Dawley) were carefully isolated, with fat and connective tissue removed. The aortas were then incised and cut into small fragments, approximately 1 to 2 mm in size. These fragments were positioned lumen-side down in culture plates and submerged in a medium composed of Dulbecco’s modified Eagle’s medium (DMEM) (Gibco, catalog no. 31966021) supplemented with 10% fetal bovine serum (FBS) (PAN-Biotech, catalog no. P40-39500) and 1% penicillin/streptomycin (P/S) (Gibco, catalog no. 15140122), to promote vascular smooth muscle cell (VSMC) migration onto the plate surface. After approximately 5 days, when VSMCs had proliferated and adhered to the plate, the aortic tissue pieces were discarded, and the cells were cultured in fresh medium until they reached confluence. VSMCs were maintained at 37°C in a 5% CO_2_ humidified incubator and were regularly passaged. Rat aortic smooth muscle cells were passaged every 2 to 3 days when confluency reached >80% and cultured in DMEM media as above.

### Isolation and culture of neonatal rat ventricular myocytes

Primary cultured cardiac ventricular myocytes were isolated from 1- to 2-day-old Sprague-Dawley rat pups (Charles River). Hearts were minced into ∼1 mm^3^ pieces in ice-cold ADS solution [NaCl (6.8 g/liter), Hepes (4.76 g/liter), NaH_2_PO_4_ (0.12 g/liter), glucose (1 g/liter), and KCl (0.4 g/liter)] and digested in collagenase (0.82 g/liter; Worthington, catalog no. LS004176) and pancreatin (0.6 g/liter; Sigma-Aldrich, catalog no. SLCD9444) at 37°C with agitation (200 rpm). Digestions were repeated three to five times, and supernatants were pooled in plating medium (68% DMEM media, Gibco, catalog no. 31966021; 17% Medium 199, Sigma-Aldrich, catalog no. M1264; 10% horse serum; 5% non–heat-inactivated FBS, PAN-Biotech, catalog no. P40-39500; 1% P/S, Gibco, catalog no. 15140122; 2% 200 mM glutamine; and 1% nonessential amino acids). Undigested tissue was cleared by a cell strainer and cells were recovered by centrifugation (1150 rpm, 5 min), followed by preplating in T175 flasks for 2 hours to remove nonmyocytes. The neonatal rat ventricular myocyte–rich supernatant was seeded onto gelatin (Sigma-Aldrich, catalog no. G1393)–coated 24-well plates and incubated at 37°C. On day 2, plating medium (DMEM/M199, 1% P/S, 2% glutamine) was replaced with maintenance medium (68% DMEM media, 17% Medium 199, 10% horse serum, 5% non–heat-inactivated FBS, 1% P/S, Gibco, 2% 200 mM glutamine, and 1% nonessential amino acids), and cells were cultured for 72 hours before treatments.

### Cell treatment

Media was changed to Earle’s Balanced Salt Solution (Gibco, catalog no. 24010043) before cells were treated with 50 μM quercetin (Sigma-Aldrich, catalog no. 117-39-5), 50 μM fisetin (Universal Bio, catalog no. CS-7840), 50 μM retinol (Sigma-Aldrich, catalog no. R7632), 50 μM retinoic acid (Sigma-Aldrich, catalog no. R2625), 50 μM urolithin A (Sigma-Aldrich, catalog no. 1143-70-0), or 50 μM ergothioneine (Sigma-Aldrich, catalog no. 497-30-3). Cells were supplemented with 1000× stocks of natural compounds, with vehicle (DMSO, Sigma-Aldrich, catalog no. D2438) also given at an equal volume to control wells. To target the expression of PKGI, cells were transfected with 50 nM PKGI siRNA (Horizon Discovery, catalog no. L-085799-02-0005) using Lipofectamine 3000 Reagent (Invitrogen, catalog no. L3000008).

### Immunoblotting

Protein samples for electrophoresis were prepared in sample buffer (SB) (50 mM tris-HCl, pH 6.8, 2% w/v SDS, 10% glycerol, and 0.0025% w/v bromophenol blue, with or without 100 mM maleimide). For nonreducing conditions, SB was supplemented with 100 mM maleimide, whereas for reducing conditions, 5% β-mercaptoethanol was added. A total of 12 μl of each sample was loaded onto 15-well Mini-PROTEAN TGX Gels (Bio-Rad, catalog no. 456-1086), then resolved at a constant 180 V. Once the dye front had reached the bottom of the cassette, gels were removed from their cases. Using semi-dry transfer (Trans-Blot Turbo Transfer System, Bio-Rad), proteins were blotted onto polyvinylidene fluoride (PVDF) membranes (Bio-Rad). Subsequently, the PVDF membranes were blocked with 10% nonfat dry milk or bovine serum albumin in phosphate-buffered saline (PBS) with 0.1% v/v tween (PBS-T) for 1 hour, then incubated with the indicated primary antibody overnight at 4°C. After primary antibody incubation, membranes were washed with PBS-T three times for 10 min each, then incubated with the appropriate horseradish peroxidase (HRP)–linked secondary antibody for 1 hour at room temperature, followed by three washes with PBS-T as before. Samples were immunoblotted for PKGIα (Enzo Life Sciences, catalog no. ADI-KAP-PK005-D), Phospho-VASP (Ser^239^) (Cell Signaling, catalog no. 3114), Phospho-PLN (Ser^16^) (Badrilla, catalog no. A010-12AP), ANP (Proteintech, catalog no. 27426-1-AP), a/b-tubulin (Cell Signaling, catalog no. 2148), or GAPDH (Cell Signaling, catalog no. 2118).

### Assays using recombinant PKGIα

The activity of recombinant PKGIα (MRC PPU, catalog no. DU26299) in response to urolithin A was measured using the PKGI Kinase Enzyme System (Promega, catalog no. V4248) and the ADP-Glo Kinase Assay (Promega, catalog no. V9101). PKGIα was prepared following the manufacturer’s instructions, excluding the addition of dithiothreitol (DTT). Urolithin A (100 μM) or 50 μM cGMP was added to the corresponding wells of a 96-well plate. Reaction buffer containing the ATP/substrate mix was then added to each well, and the mixture was incubated for 60 min at room temperature. ADP-Glo Reagent was subsequently added to deplete residual ATP, followed by a 40-min incubation at room temperature. Kinase Detection Reagent was next added to detect ADP, and after an additional 60-min incubation, luminescence was measured using a GloMax Discover Microplate Reader (Promega, catalog no. GM3000). In some experiments, 10 ng of recombinant PKGIα was treated with or without 10 mM DTT for 20 min, followed by desalting using Zeba Spin Desalting Columns (Thermo Fisher Scientific, catalog no. 10056033). Samples were then incubated with or without 100 μM diamide in the presence or absence of urolithin A for 10 min, after which sample buffer containing maleimide was added.

### Myography

Mouse mesenteric vessels were dissected and isolated, and rings were cut into ∼3-mm lengths in prewarmed Krebs buffer (118 mM NaCl, 2.5 mM CaCl_2_, 11 mM glucose, 25 mM NaHCO_3_, 4.7 mM KCl, 1.18 mM KH_2_PO_4_, and 1.16 mM MgSO_4_). The vessel rings were mounted with a 25-μm wire in a Multi Wire Myograph System (DMT 620M) filled with Krebs buffer maintained at 37°C and bubbled with mixed gas (95% O_2_, 5% CO_2_). Optimal pre-tension was applied using the system’s normalization feature. After a 60-min equilibration, the vessels were activated with 60 mM KCl to induce contraction, followed by three washes. After a 15-min resting period, 10^−7^ M U46619 was used to induce contraction. In some experiments, vessels were preincubated with 5 μM ODQ for 3 hours. Following preincubation with U46619, cumulative half-log dose-response curves for fisetin, quercetin, or urolithin A (10^−7^ to 10^−4^ M) were applied to WT or C42S PKGIα KI mesenteric vessels.

### mBBr assay

Reactions were prepared with or without 2 mM urolithin A or 2 mM quercetin, in the presence or absence of 1 mM cysteine, in 100 mM tris buffer (pH 8). After gentle mixing, samples were incubated at 37°C for 1 hour. Following incubation, 2 mM mBBr (ChemCruz, catalog no. sc-214629) was added to each sample and the reactions were incubated for a further 1 hour at 37°C. mBBr reacts with thiols to form a fluorescent adduct, which was measured using a microplate reader (SpectraMax GeminiXS, Molecular Devices) at an excitation wavelength of 394 nm and an emission wavelength of 490 nm.

### Liquid chromatography–tandem mass spectrometry

Recombinant PKGIα (20 μg, MRC PPU, catalog no. DU26299) was treated with vehicle or 2 mM urolithin A for 1 hour. After incubation, each sample was buffer exchanged into 8 M urea buffer using a 3-kDa molecular weight cutoff filter (Amicon ultra, 0.5 ml) and spun at 14,000 rpm at 4°C. After the buffer exchange, proteins were digested within the filter using trypsin with a ratio of 1:100 (enzyme:protein) at 37°C overnight. After acidifying the enzyme digestion with 1% acetonitrile and 0.1% trifluoroacetic acid, peptides were collected by spinning down the filter, desalted with a C18 column, and dried in a speedVac before liquid chromatography–tandem mass spectrometry analysis.

Samples were analyzed using an Orbitrap Eclipse Tribrid mass spectrometer (Thermo Fisher Scientific) with data search against the sequence of recombinant PRKG1 isoform 1 (MRC PPU, catalog no. DU26299) using Thermo Proteome Discoverer (PD, version 2.5), with standard tryptic parameters and dynamic modifications for methionine oxidation and cysteine modification by urolithin A. Peptide modifications that met the false discovery rate cutoff of 1% were accepted only with ptmRS site probability equal to 100.

### Animal studies

Adult animals were euthanized under terminal anesthesia induced by an intraperitoneal injection of sodium pentobarbital (250 mg/kg, Pentoject) combined with sodium heparin (1000 IU/kg) to ensure deep anesthesia and anticoagulation. Adequacy of anesthesia was confirmed by the absence of corneal and pedal reflexes. Following terminal anesthesia, death was confirmed by cervical dislocation. Neonatal animals were not subjected to any prior interventions and were used solely for tissue or cell isolation.

For surgical procedures, animals were anesthetized using inhaled isoflurane (Covetrus) delivered in oxygen (induction at 5%, maintenance at 1.5 to 2%) via a nose cone. Depth of anesthesia was monitored continuously by assessing respiratory rate and corneal and pedal withdrawal reflexes. Body temperature was maintained using a thermostatically controlled heating pad. Analgesia was provided according to the following protocol: 30 min before surgery, animals received buprenorphine (0.1 mg/kg, subcutaneously, Vetergesic) and meloxicam (5 mg/kg, subcutaneously, Metacam) for preoperative analgesia. For postoperative analgesia, buprenorphine (0.1 mg/kg, subcutaneously, Vetergesic) was administered for up to 2 days, and meloxicam (5 mg/kg, subcutaneously, Metacam) was administered for up to 5 days, depending on the animal’s clinical condition.

All procedures were performed in accordance with the UK Home Office Guidance on the Operation of the Animals (Scientific Procedures) Act 1986. Sample sizes were determined based on previous studies, and all animal experiments were approved by the King’s College London Animal Welfare and Ethical Review Body. Mice used in this study were on the C57BL/6J genetic background. Male C42S PKGIα KI mice or WT littermate controls were used in studies at 12 to 15 weeks of age. Mice constitutively expressing the PKGIα C42S mutation were generated on a pure C57BL/6 background by TaconicArtemis GmbH as previously described (*5*). No animals were excluded from this study.

### Mouse model of HFpEF

The DOCA-salt hypertension model was used as it leads to diastolic dysfunction (*48*). However, we used a modification to this model where HF diet was included to induce a more robust and pathologically relevant model of HFpEF. The unilateral nephrectomy was performed, followed by subcutaneous implantation of a DOCA pellet (Innovative Research of America, catalog no. M-121). On the day of DOCA-salt administration and unilateral nephrectomy, mice were provided with drinking water containing 1% NaCl and the standard diet was replaced with an HF diet (SAFE, catalog no. 292HF, 60% calories from lipids). At the same time, mice were singly housed in cages equipped with running wheels. The Sham group underwent surgical procedures but without nephrectomy, DOCA implantation, NaCl supplementation, or HF diet. Twenty-one days after starting the HF diet and DOCA-salt administration, mice were also administered urolithin A (50 mg/kg per day, dissolved in 0.25% CMC-Na) or vehicle control (0.25% CMC-Na) daily for 7 days by oral gavage. Mice of both genotypes were randomly subdivided into two groups per condition, resulting in the establishment of six distinct experimental groups: (i) WT Sham; (ii) WT HFpEF; (iii) WT HFpEF + urolithin A; (iv) C42S PKGIα KI Sham; (v) C42S PKGIα KI HFpEF; and (vi) C42S PKGIα KI HFpEF + urolithin A. At the end of the protocol, hearts were harvested and the blood in the chamber was gently removed with tweezers. The heart weight and the tibia length were then measured. The normalized heart weight was calculated by dividing the heart weight by tibia length. Tissues were then flash frozen in liquid nitrogen for subsequent analysis.

### Echocardiography

Echocardiography was performed using high-frequency linear array transducers (MX400, 30 MHz, axial resolution: 50 μm, lateral resolution: 110 μm) equipped with a Vevo 3100 high-resolution imaging system (FUJIFILM VisualSonics, Canada). Mice were anesthetized with 1.5% isoflurane to achieve constant and comparable heart rates during image acquisition and imaged in the supine position on an electrical heating pad to maintain the core temperature around 37°C. Electrocardiographic monitoring was carried out by limb electrodes. Subsequently, a standardized two-dimensional echocardiographic examination was performed to evaluate left ventricular EF and FS. Pulsed wave Doppler flow and Tissue Doppler imaging were obtained in the apical four-chamber view. All perspectives were archived as cine loops encompassing 300 frames.

The cardiac function was measured by echocardiography before DOCA administration, and 21 and 28 days after the implantation. All captured images were archived in raw DICOM format for subsequent offline analyses using the Vevo3100 LAB Software 5.7.1 (FUJIFILM VisualSonics, Canada). Diastolic parameters, including average rLSR, IVRTc, *E*/*e*′ ratio, *E*/*A* ratio, and left ventricular GLS analysis, were acquired and evaluated as described previously (*10*). All images were analyzed offline with blinding of mouse experimental groups.

### Histopathological analysis

Mouse hearts were excised, and the ventricular tissues were fixed overnight in 4% paraformaldehyde. Samples were then embedded in paraffin and sectioned into 5-μm-thick slices. Sections were dewaxed and stained with wheat germ agglutinin Alexa Fluor 488 conjugate (Invitrogen, catalog no. W11261). Stained heart tissues were observed using a confocal microscope system (Leica TCS-SP5). Myocardial fibrosis was assessed using Masson’s trichrome staining kit (Abcam, catalog no. ab150686) following the manufacturer’s instructions.

### Human-derived EHT

Cardiomyocytes were differentiated from the KOLF2 human induced pluripotent stem cell line using the StemMACS CardioDiff Kit (Miltenyi Biotec, catalog no. 130-125-28), according to the manufacturer’s instructions. Cells exhibiting wave-like contractions were dissociated from day 10 onward. Further experiments were performed at 14 to 25 days after the initiation of differentiation.

Fibrin-based human EHTs were then generated as previously described (*49*). Casting molds were prepared in a 24-well plate format by using 2% agarose in PBS and a polytetrafluoroethylene (PTFE) spacer (EHT-Technologies, catalog no. C0002), forming a mold for EHT embedding. After agarose solidification, transparent polydimethylsiloxane (PDMS) posts (EHT-Technologies, catalog no. C0001) were inserted into the molds. To generate each EHT, 1 million cardiomyocytes were mixed with 5.6 μl of 2× DMEM, 0.1% Y-27632 (10 mM; Sigma-Aldrich, catalog no. Y0503), 2.5 μl of fibrinogen (200 mg/ml in 0.9% NaCl; Sigma-Aldrich, catalog no. F8630), and 97 μl of NKM medium (DMEM, Sigma-Aldrich, catalog no. D5796; 10% FBS, Sigma-Aldrich, catalog no. F9665; 1% P/S, Gibco, catalog no. 11548876; and 1% l-Glutamine, Sigma-Aldrich, catalog no. G7513). Then, 3 μl of thrombin (Sigma-Aldrich, catalog no. T4648) was added to the master mix, and immediately after mixing, was pipetted into each agarose mold. The plate was incubated at 37°C with 7% CO_2_, 40% O_2_, and 98% relative humidity for 1.5 to 2 hours to facilitate fibrinogen polymerization. After polymerization, 500 μl of prewarmed NKM medium was added to overlay the EHTs for 30 min. EHTs were then carefully transferred to a new 24-well plate containing prewarmed EHT culture medium [DMEM; 10% horse serum, Sigma-Aldrich, catalog no. H1138; 1% P/S; and insulin (10 μg/ml), Sigma-Aldrich, catalog no. I0516]. Medium was changed three times per week by transferring the EHTs with their posts into 1.5 ml of fresh, prewarmed EHT medium.

Urolithin A (100 μM) or a vehicle control was added to EHTs. Measurements were conducted during spontaneous activity at three time points: before treatment, and 30 and 120 min after treatment. Contraction analysis of the EHTs was performed using the MuscleMotion software, focusing on contraction force and kinetics (*50*). The contraction kinetics were assessed by measuring contraction and relaxation times at 90, 50, and 20% of peak tension (*T*_90%_, *T*_50%_, and *T*_20%_, respectively).

The contractile force (*F*) was calculated based on the postdeflection degree (δ, μm), the silicone post length (*L*), radius (*R*), and the elastic modulus (*E*) of the PDMS. The calculation followed the formula (*51*): $F=\frac{3\pi ER^{4}\delta }{4L^{3}}$.

### Statistical analysis

All measurements were performed using at least three independent biological replicates from separate experiments, and no data were excluded. Data are shown as mean ± SEM. The exact sample size and statistical test used for each experiment are specified in the corresponding figure legends. Differences between groups were assessed using analysis of variance (ANOVA), followed by a two-tailed unpaired Student’s *t* test or, for multiple comparisons, Tukey’s post hoc test. Results were considered significant at a 5% significance level.
