# Supplementary material for: Targeting PKGIα Cys42 attenuates cardiac dysfunction in heart failure with preserved ejection fraction
Source: Sci Adv. 2026 Aug 19;12(34):eaec8088. doi: 10.1126/sciadv.aec8088 (PMC13488924; doi:10.1126/sciadv.aec8088)
Supplement: Supplementary file 1 [file sciadv.aec8088_sm.pdf]

Science Advances
AAAS

Supplementary Materials for

Targeting PKGIα Cys^{42} attenuates cardiac dysfunction in heart failure with preserved ejection fraction

Jie Su *et al.*

Corresponding author: Joseph R. Burgoyne, joseph.burgoyne@kcl.ac.uk

Sci. Adv. **12**, eaec8088 (2026)
DOI: 10.1126/sciadv.aec8088

This PDF file includes:

Figs. S1 to S3

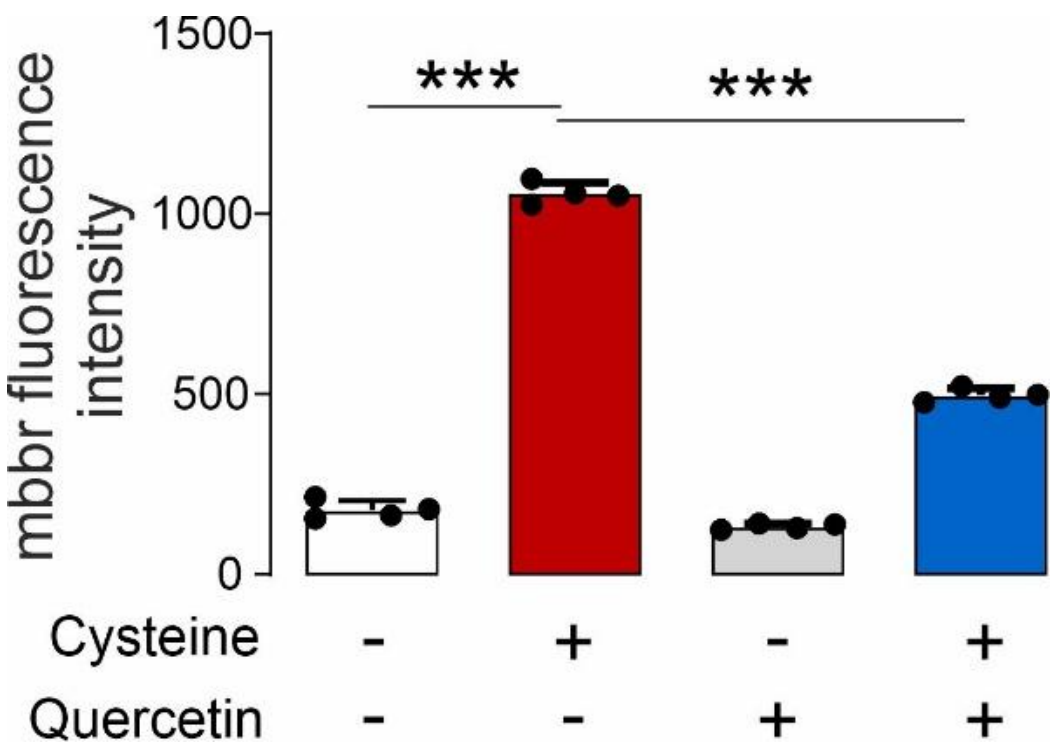

Fig. S1. Monobromobimane (mbbr) assay assessing the modification of cysteine by quercetin (n=4).

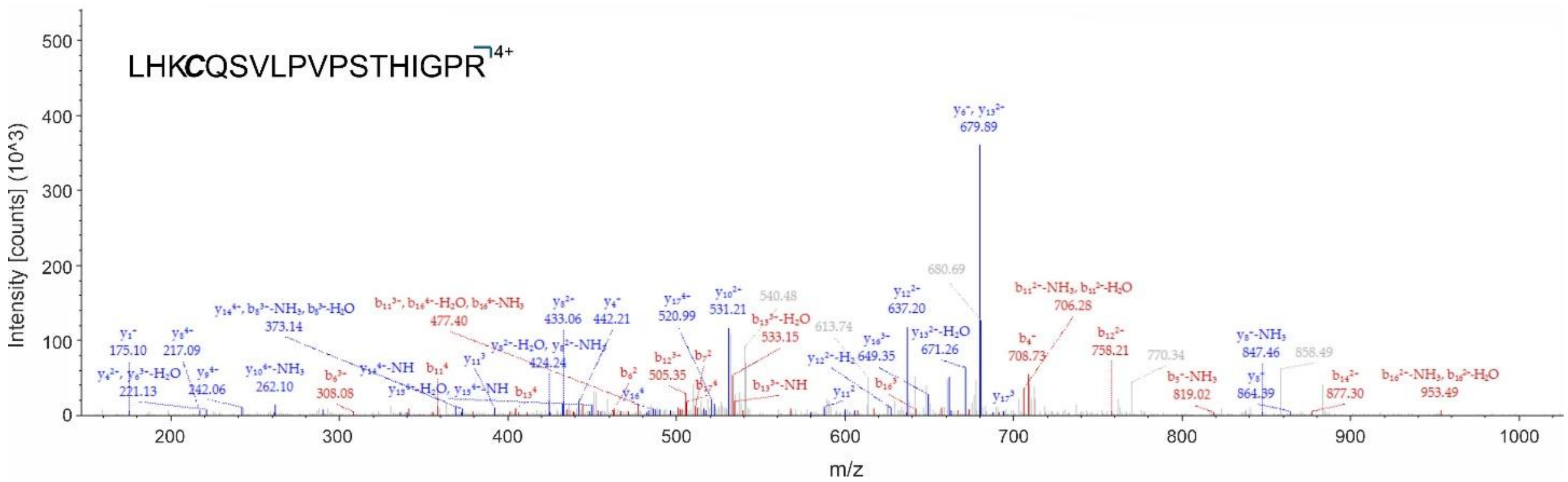

Fig. S2. Mass spectrometry analysis of PKG1α revealed a fragmentation pattern confirming the modification of C42 by urolithin A

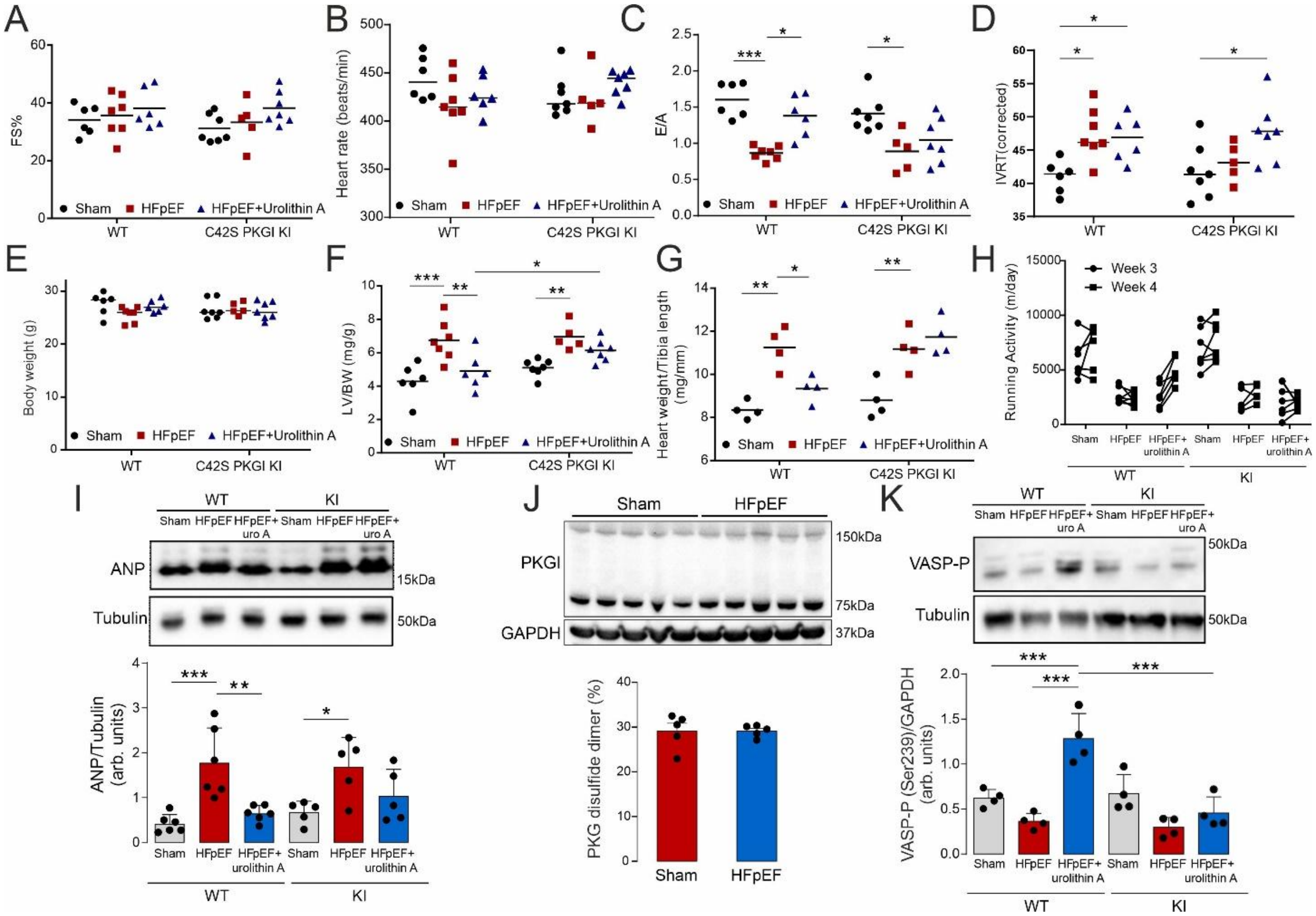

Fig. S3. Assessing (**A**) Heart rate, (**B**) Left ventricular fractional shortening (FS), (**C**) E/A ratio, (**D**) heart rate-corrected isovolumic relaxation time (IVRTc). (**E**) body weight at 4 weeks, (**F**), left ventricular weight/body weight (LV/BW) (n=5-7) and (**G**), heart weight/tibia length at 4 weeks after sham surgery, HFpEF, or HFpEF with one week of diet supplementation with urolithin A (n=4). (**H**) Running activity in mice at 3 and 4 weeks after sham surgery, HFpEF, or HFpEF with one week of diet supplementation (n=5-7). (**I**) ANP in heart tissue from WT or C42S PKGIα KI mice after sham surgery, HFpEF, of HFpEF with one week of diet supplementation with urolithin A (n=6-7). (**J**) PKGIα disulfide dimerization in heart tissue from WT mice after sham surgery or HFpEF at three weeks (n=5). (**K**) VASP phosphorylation in heart tissue from WT or C42S PKGIα KI mice after sham surgery, HFpEF, of HFpEF with one week of diet supplementation with urolithin A (n=4). *P<0.05; **P<0.01; ***P<0.005. Comparisons were made using 2-way ANOVA followed by the Tukey post hoc test.
